# The impact of comorbidity status on knee function 1 year after total knee arthroplasty: a population-based cohort study

**DOI:** 10.2340/17453674.2024.40706

**Published:** 2024-05-17

**Authors:** Katrine Glintborg IVERSEN, Rikke Sommer HAABER, Martin Bækgaard STISEN, André Sejr KLENØ, Martin LINDBERG-LARSEN, Alma Becic PEDERSEN, Inger MECHLENBURG

**Affiliations:** 1 Department of Clinical Epidemiology, Aarhus University Hospital, Aarhus; 2 Department of Orthopaedic Surgery, Aarhus University Hospital, Aarhus; 3 Department of Clinical Medicine, Aarhus University, Aarhus; 4 Department of Orthopaedic Surgery, Odense University Hospital; 5 University of Southern Denmark, Odense, Denmark

## Abstract

**Background and purpose:**

Few studies have examined the impact of comorbidity on functional and clinical knee scores after primary total knee arthroplasty (TKA). We compared the effect of having a high Charlson Comorbidity Index (CCI), relative to a low CCI, on changes in the American Knee Society Score (AKSS) functional and clinical scores from baseline to week 52 after TKA in patients with knee osteoarthritis (OA).

**Methods:**

This population-based cohort study included 22,533 patients identified in the Danish Knee Arthroplasty Register from 1997 to 2021. Patients were classified as having low, medium, or high comorbidity based on CCI. The outcome was defined as the mean change (from preoperative to 1-year post-TKA) in functional and clinical knee scores measured by the AKSS (0–100). The association was analyzed using multiple linear regression by calculating mean change scores adjusting for sex, age, weight, cohabiting status, and baseline AKSS.

**Results:**

The prevalence of patients with low, medium, and high comorbidity was 75%, 21%, and 4%, respectively. The mean change score in functional AKSS for patients with high comorbidity was –6 points (95% confidence interval [CI] –7 to –5) compared with low comorbidity. The mean change score in clinical AKSS for patients with high comorbidity was –1 point (CI –2 to 0) compared with low comorbidity.

**Conclusion:**

Patients with knee OA and medium or high comorbidity can expect similar improvements in functional and clinical AKSS after TKA to patients with low comorbidity.

Worldwide, comorbidity is an increasing concern due to the demographic development with aging populations [1]. Comorbidity is associated with significant functional impairment, more severe pain after surgery, and poorer pain management [2].

Several studies report an association between comorbidity and functional impairment and knee pain after total knee arthroplasty (TKA), but they are limited by small and heterogeneous samples [3-6]. Hence, we aimed to investigate the impact of comorbidity on changes in functional and clinical knee scores following TKA. The secondary aim was to present baseline values in functional impairments and knee pain in patients with OA undergoing TKA categorized according to the severity of comorbidity.

## Methods

### Study design and population

This Danish population-based cohort study was conducted in patients aged above 18 years with OA undergoing primary TKA between January 1997 and September 2021, identified in the Danish Knee Arthroplasty Registry (DKR).

This study retrieved relevant data from the following registries: the DKR, the Danish Civil Registration System (CRS), the Danish National Patient Registry (DNPR), and Statistics Denmark (DST).

The date of primary TKA in the DKR was considered the index date. The DKR contains information on TKA surgeries from all public orthopedic departments and private hospitals in Denmark [7]. The CRS contains information on all citizens in Denmark. Every citizen in Denmark is assigned a unique 10-digit personal identification number at birth, which is used in all Danish medical registers [8]. This allows for complete individual-level linkage of data across Danish registers. The CRS contains information on age and sex. The DNPR contains among other information on all hospital, outpatient clinic, and emergency room visits. Diagnoses were classified according to the International Classification of Diseases (ICD 8 and 10) [9]. Lastly, all data for analysis in this study are hosted at DST, including data on participants’ cohabiting status.

### Comorbidity

Comorbidity is defined as “Any distinct additional entity that has existed or may occur during the clinical course of a patient who has the index disease under study” [10], and this definition is widely adopted in the scientific literature. We included data on comorbidity measured by the Charlson Comorbidity Index (CCI), which categorizes comorbidity based on ICD codes [11]. Based on the weighted index (0–37 points), the CCI was categorized as; low = a score of 0, medium = a score of 1 or 2, and high = a score of 3 or more [11]. To estimate a patient’s CCI burden, we used a 10-year lookback period from the index date.

### American Knee Society Score (AKSS)

The outcomes in this study were changes in the AKSS of both functional and clinical knee scores measured as the difference between baseline (preoperatively) and at 1-year follow-up score after primary TKA.

The AKSS is divided into 2 components: a functional knee score and a clinical knee score. The functional knee score assesses functional impairment by the patient’s ability to walk, climb stairs, and use of a walking aid. The clinical knee score rates knee stability, range of motion, malalignment, and pain [12]. The AKSS is a valid, reliable, and widely used functional outcome score (0–100, 100 best) for patients undergoing TKA [13,14]. A change score from baseline to 1-year follow-up at 9.2 (95% confidence interval [CI] 7.3–10.2) points in the functional knee score and 7.2 (CI 5.1–7.8) points in the clinical knee score is considered a minimal clinically important difference (MCID) [15]. At the same time, threshold values for treatment success after TKA are 72.2 points for functional knee score and 85.5 points for clinical knee score [16]. Data in the AKSS are retrieved from the DKR, but registration is not mandatory; in consequence the completeness of the registration in the study period is 22%. The same version of AKSS was used throughout the study period.

### Covariates

To account for potential confounding factors influencing the association between comorbidity and both functional and clinical AKSS, we considered age, sex, weight, cohabiting status, and baseline AKSS scores. These are individual predictors of the outcome and are not intermediate factors. Moreover, previous studies have adjusted for the same variables [4,6].

Age was categorized into < 56, 56–65, 66–75, and > 75 years. Cohabiting status was reported as living alone, cohabiting (married or living as a couple), or other (e.g., households with multiple families) [17]. Furthermore, body mass index (BMI) is considered as a potential confounder influencing the association. Due to limited access to BMI data for the period between 2011 and 2021, we included patients’ weight in the regression analyses instead and used BMI in the descriptive data only. BMI was categorized into 3 groups: under and normal weight ( < 25), pre-obese (25–29.9), and obese (≥ 30). Under and normal weight were aggregated into a single category due to the small number of underweight patients in the sample. Weight is presented as a continuous variable.

### Statistics

Patient characteristics and baseline means, by CCI group and overall, were summarized for primary TKA with frequencies (n) and percentages (%) at the index date and calculated a standardized difference (std diff) indicating whether there were significant differences between CCI low and CCI high. The std diff was calculated for both the dataset and missing data. We interpreted a std diff of ≥ 0.2 as an indication of a difference. We presented 1-year follow-up means, and changes in functional and clinical AKSS by CCI group. Distribution of all continuous variables was assessed for normality. A simple linear regression was performed to examine differences in mean changes in functional and clinical AKSS between patients with or without comorbidity as a crude estimate. We analyzed associations between comorbidity and mean changes in functional and clinical AKSS by multiple linear regression analyses, adjusting for the sex, age, weight, cohabitating status, and baseline AKSS. The assumptions of simple and multiple linear regression analyses were based on plots of observed versus predicted values, scatter plots, residual plots, histograms, and QQ plots. The results of the analyses were presented as coefficients for each CCI group with CI.

The primary analysis was based on patients with complete AKSS data at baseline and at 1-year follow-up and covariates (sex, age, weight, and cohabiting). We looked for differences in demographics between those who were included in the study and those who were not due to missing AKSS to test whether data was missing at random. The significance level was set at α = 0.05. All analyses were performed by StataBE17 (StataCorp, College Station, TX, USA).

### Ethics, funding, data sharing, and disclosures

According to Danish law, ethics approval is not needed for register-based studies. The study was reported to the Danish Data Protection Agency (Aarhus University record number 2016-051-000001, id. nr. 880). As a part of the Data Use Agreement at the DKR and the DST, authors are not allowed to provide raw data. This research was partly funded by the Orthopedic Research Foundation, Aarhus University Hospital. The authors declared no conflicts of interest. Complete disclosure of interest forms according to ICMJE are available on the article page, doi: 10.2340/17453674.2024.40706

## Results

### Study population

**Figure 1:**
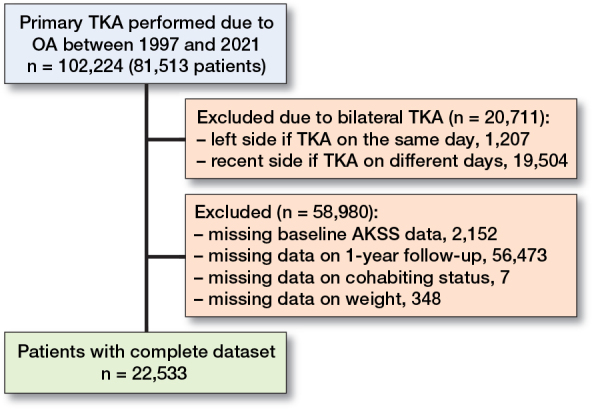
Flowchart illustrating the inclusion and exclusion process of the study population.TKA = total knee arthroplasty. OA = osteoarthritis. AKSS = American Knee Society Score.

We included 81,513 patients with OA undergoing a total of 102,224 TKAs. As some patients had a bilateral TKA on the same day or on different dates, 20,711 operations were excluded. If bilateral operations were performed on the same day, data from the left knee was excluded. Otherwise, the first operation remained in the data set. 2,152 patients were excluded due to missing data on baseline AKSS. Furthermore, a total of 56,828 patients were excluded due to missing 1-year follow-up AKSS or covariate data (Figure 1). The final study population encompassed 22,533 patients, equivalent to 22% of patients. Patients were predominantly female (64%), cohabiting (57%), and had no comorbidities at index date (75%). The mean age at index date was 69 years with a standard deviation (SD) of 9 (Table 1).

**Table 1:** Patient characteristics by CCI groups in patients undergoing primary TKA due to OA at index date. Values are count (%) unless otherwise specified

Factor	Overall	CCI low	CCI medium	CCI high	std diff^a^
Overall	22,533 (100)	16,904 (75)	4,641 (21)	988 (4)	
Sex					0.19
Female	14,304 (64)	10,909 (65)	2,848 (61)	547 (56)	0.19
Male	8,229 (36)	5,995 (35)	1,793 (39)	441 (44)	0.19
Age^b^, mean (SD)	69 (9)	68 (9)	70 (9)	72 (8)	–0.38
Age groups					0.38
< 56	1,852 (8)	1,555 (9)	268 (6)	29 (3)	0.26
56–65	6,032 (27)	4,743 (28)	1,099 (24)	190 (19)	0.21
66–75	8,945 (40)	6,533 (39)	2,986 (43)	426 (43)	0.09
> 75	5,704 (25)	4,073 (24)	1,288 (28)	343 (35)	0.23
Cohabiting status					0.09
Alone	8,569 (38)	6,366 (38)	1,790 (39)	413 (42)	0.08
Cohabiting	12,825 (57)	9,658 (57)	2,643 (57)	524 (53)	0.08
Other	1,139 (5)	880 (5)	208 (4)	51 (5)	0.002
Weight, mean (SD)	84 (18)	83 (18)	84 (17)	85 (17)	–0.12
BMI^c^					0.04
Underweight and normal	1,293 (20)	892 (20)	329 (20)	72 (19)	0.08
Pre-obese	2,518 (39)	1,742 (39)	621 (38)	155 (40)	0.16
Obese	2,678 (41)	1,827 (41)	690 (42)	161 (41)	0.16
Baseline AKSS, mean (SD)					
Clinical score	34 (17)	34 (17)	34 (17)	34 (17)	0.34
Functional score	48 (18)	49 (17)	47 (18)	43 (19)	–0.01

CCI = Charlson Comorbidity Index. TKA = total knee arthroplasty. OA = osteoarthritis. SD = standard deviation. BMI = body mass index. AKSS = American Knee Society score. st diff = standardized difference.

a Between CCI low and CCI high.

b Date of primary TKA is considered index date.

c Based on 6,489 patients in the Danish Knee Arthroplasty Register 2011–2021.

### Missing data analysis

The missing data analysis showed that patients without complete data on AKSS had the same mean age of 69 years (SD 10) as the study population. Patients were still predominantly female (61%), cohabiting (57%), and had no comorbidities at index date (74%) (Tables 2 and 3, see Appendix).

**Table 2:** Patient characteristics by CCI groups of the patients undergoing primary TKA due to OA with missing data on AKSS in 1-year follow-up. Values are count (%) unless otherwise specified

Factor	Overall	CCI low	CCI medium	CCI high	std diff^a^
Overall	56,828 (100)	41,976 (74)	11,838 (21)	3,014 (4)	
Sex					0.17
Female	34,854 (61)	26,233 (63)	6,993 (59)	1,628 (54)	0.17
Male	21,974 (39)	15,743 (37)	4,845 (41)	1,386 (46)	0.17
Age^,^ mean (SD)^b^	69 (10)	68 (10)	70 (9)	72 (8)	–0.38
Age groups					0.39
< 56	5,279 (9)	4,390 (10)	782 (7)	107 (4)	0.27
56–65	14,425 (25)	11,133 (27)	2,764 (23)	528 (17)	0.22
66–75	22,429 (40)	16,205 (39)	4,903 (41)	1,321 (44)	0.11
> 75	14,695 (26)	10,248 (24)	3,389 (29)	1,058 (35)	0.24
Cohabiting status^c^					0.15
Alone	19,960 (35)	14,231 (34)	4,477 (38)	1,252 (42)	0.16
Cohabiting	32,339 (57)	24,203 (58)	6,566 (55)	1,570 (52)	0.11
Other	4,173 (7)	3,186 (8)	795 (7)	192 (6)	0.04
Weight, mean (SD)^d^	84.5 (18)	84.2 (18)	85.1 (18)	86.0 (18)	–0.10
BMI^e^					0.04
Underweight and normal	7,276 (20)	5,331 (20)	1,558 (20)	387 (19)	0.004
Pre-obese	14,428 (40)	10,613 (40)	3,034 (39)	781 (39)	0.01
Obese	14,581 (40)	10,629 (40)	3,102 (41)	850 (42)	0.07
Baseline AKSS, mean (SD)					
Clinical score	33 (17)	33 (17)	33 (17)	33 (17)	0.22
Functional score	48 (19)	49 (19)	47 (19)	44 (20)	0.26

For abbreviations, see Table 1.

a Between CCI low and CCI high.

b Date of primary TKA is considered index date.

c Based on 41,620 in CCI low, 11,838 in CCI medium, 3,014 in CCI high

d Based on 40,988 in CCI low, 11,460 in CCI medium, 2,912 in CCI high.

e Based on 26,573 in CCI low, 7,694 in CCI medium, 2,018 in CCI high.

**Table 3:** Patient characteristics of the patients undergoing primary TKA due to OA with any missing data including missing AKSS in both or either baseline or 1-year follow-up (N = 58,980). Values are count (%) unless otherwise specified

Sex	
Female	36,176 (61)
Male	22,804 (39)
Age at TKA, mean (SD)	69 (9)
Age groups	
< 56	5,446 (9)
56–65	14,982 (25)
66–75	23,268 (39)
> 75	15,284 (26)
Charlson Comorbidity Index	
Low (0)	43,466 (74)
Medium (1–2)	12,385 (21)
High (≥ 3)	3,129 (5)
Cohabiting status^a^	
Alone	20,764 (35)
Cohabiting	33,554 (57)
Other	4,305 (7)
Weight, mean (SD)	85 (18)
BMI^b^	
Underweight and normal	7,375 (20)
Pre-obese	14,618 (40)
Obese	14,785 (40)

For abbreviations, see Table 1.

a Based on 58,275 patients.

b Based on 36,778 patients.

### Baseline and follow-up scores in functional and clinical AKSS for different CCI groups

In functional knee scores, the overall mean baseline score decreased from 49 points (SD 17) in the CCI-low group to 47 points (SD 19) in the CCI-medium group, to 44 points (SD 20) in the CCI-high group (Table 1). The same tendencies were seen in the mean 1-year follow-up score, with 78 points (SD 21) in the CCI-low, 75 points (SD 23) in the CCI-medium, and 69 points (SD 25) in the CCI-high group, respectively (Table 4). In the clinical knee score, the overall mean baseline score was 34 points (SD 17) in all 3 CCI groups (Table 1). The mean 1-year follow-up was 82 points (SD 17), 83 points (SD 17), and 82 points (SD 18) in the CCI low, medium, and high groups, respectively (Table 4).

**Table 4:** 1-year follow up and change in functional and clinical AKSS in patients undergoing primary TKA due to OA

Factor	CCI low		CCI medium		CCI high		Mean change differences between strata			
	1-year FU	change	1-year FU	change	1-year FU	change	CCI low vs CCI medium		CCI low vs CCI high	
	mean (SD)	(CI)	mean (SD)	(CI)	mean (SD)	(CI)	mean (CI)	P value	mean (CI)	P value
**Functional AKSS**										
Overall	78 (21)	29 (29–29)	75 (23)	28 (27–29)	69 (25)	26 (25–28)	1.2 (0.5 to 1.9)	< 0.001	2.6 (1.2 to 4.0)	< 0.001
Sex										
Female	75 (21)	28 (28–29)	71 (23)	26 (25–27)	65 (25)	25 (23–27)	2.0 (1.1 to 3.0)	1	3.1 (1.1 to 5.0)	0.002
Male	84 (19)	30 (30–31)	81 (21)	30 (29–31)	75 (23)	28 (26–30)	0 (–1.2 to 1.1)	1	2.3 (0.3 to 4.4)	0.03
Age groups										
< 56	81 (19)	28 (27–29)	78 (21)	26 (23–29)	72 (24)	25 (15–35)	1.7 (–1.2 to 4.5)	0.3	2.4 (–5.7 to 10.4)	0.6
56–65	82 (18)	30 (29–30)	79 (20)	29 (28–30)	74 (22)	26 (22–29)	0.9 (–0.5 to 2.3)	0.2	4.0 (0.9 to 7.1)	0.01
66–75	79 (20)	30 (29–30)	77 (22)	29 (28–30)	72 (25)	27 (25–29)	1.2 (0.1 to 2.3)	0.03	2.9 (0.8 to 5.0)	0.01
> 75	70 (23)	27 (27–28)	67 (25)	26 (25–28)	63 (25)	26 (24–29)	1.2 (–0.3 to 2.7)	0.1	1.0 (–1.5 to 3.6)	0.4
Cohabiting status										
Alone	73 (22)	28 (27–28)	69 (24)	25 (24–26)	64 (25)	25 (23–28)	2.4 (1.2 to 3.6)	0	2.4 (0.1 to 4.7)	0.04
Cohabiting	81 (19)	30 (29–30)	78 (21)	30 (29–30)	74 (23)	28 (26–30)	0.3 (–0.6 to 1.2)	0.5	2.3 (0.4 to 4.1)	0.02
Other	78 (22)	29 (28–31)	75 (23)	28 (25–31)	66 (30)	24 (17–30)	1.2 (–2.3 to 4.6)	0.5	5.6 (–0.9 to 12)	0.1
BMI^a^										
Underweight and normal	83 (19)	31 (30–32)	78 (23)	30 (28–33)	70 (26)	29 (23–34)	0.8 (–2.0 to 3.5)	0.6	2.4 (–2.7 to 7.5)	0.3
Pre-obese	83 (19)	30 (29–31)	77 (23)	29 (28–31)	72 (26)	29 (25–33)	0.7 (–1.2 to 2.6)	0.5	1.4 (–1.9 to 4.8)	0.4
Obese	77 (20)	29 (28–30)	73 (23)	28 (27–30)	70 (25)	30 (26–33)	0.4 (–1.5 to 2.3)	0.7	–0.9 (–4.3 to 2.6)	0.6
**Clinical AKSS**										
Overall	82 (17)	49 (48–49)	83 (17)	49 (48–50)	82 (18)	48 (46–50)	–0.3 (–1.1 to 0.5)	0.5	0.7 (–0.8 to 2.3)	0.3
Sex										
Female	82 (17)	48 (47–48)	82 (17)	48 (47–49)	81 (19)	48 (46–50)	–0.0 (–1.0 to 1.0)	0.9	0.1 (–2.0 to 2.2)	0.9
Male	84 (16)	50 (50–51)	85 (16)	51 (50–52)	83 (17)	48 (46–51)	–0.5 (–1.7 to 0.7)	0.4	2.1 (–0.1 to 4.3)	0.07
Age groups										
< 56	79 (19)	45 (44–47)	78 (20)	44 (41–48)	79 (22)	48 (35–60)	0.9 (–2.4 to 4.2)	0.6	–2.3 (–11.6 to 7.1)	0.6
56–65	83 (17)	48 (48–49)	82 (18)	48 (47–50)	81 (19)	46 (42–49)	0.2 (–1.4 to 1.8)	0.8	2.6 (–0.9 to 6.1)	0.1
66–75	83 (16)	49 (49–50)	84 (16)	49 (48–50)	82 (18)	48 (46–50)	0.3 (–0.9 to 1.5)	0.6	1.3 (–1.0 to 3.6)	0.3
> 75	83 (16)	50 (49–51)	84 (15)	51 (50–53)	83 (17)	50 (47–52)	–1.1 (–2.6 to 0.3)	0.1	0.5 (–2.1 to 3.1)	0.7
Cohabiting status										
Alone	82 (17)	49 (48–50)	83 (16)	49 (48–50)	82 (18)	49 (46–51)	0.0 (–1.2 to 1.3)	0.9	0.1 (–2.3 to 2.5)	0.9
Cohabiting	83 (16)	49 (48–49)	84 (17)	49 (48–50)	82 (18)	47 (45–49)	–0.5 (–1.5 to 0.6)	0.4	1.6 (–0.4 to 3.7)	0.1
Other	81 (17)	49 (47–50)	82 (18)	50 (47–53)	80 (20)	52 (44–60)	–1.1 (–4.8 to 2.7)	0.6	–3.2 (–10.3 to 3.9)	0.4
BMI ^a^										
Underweight and normal	85 (17)	49 (48–51)	87 (16)	50 (48–53)	89 (15)	55 (50–61)	–1.0 (–3.9 to 2.0)	0.5	–6.0 (–11.5 to –0.4)	0.04
Pre-obese	86 (15)	52 (51–53)	85 (17)	51 (50–53)	82 (20)	46 (42–50)	0.6 (–1.4 to 2.6)	0.6	6.1 (2.4 to 9.7)	< 0.001
Obese	83 (17)	52 (51–53)	83 (17)	51 (50–53)	85 (15)	56 (53–59)	0.7 (–1.3 to 2.7)	0.5	–3.5 (–7.1 to 0.2)	0.06

For abbreviations, see Table 1.

a Based on 6,489 patients registered in the Danish Knee Arthroplasty Register during 2011–2021.

### Changes in functional and clinical AKSS and impact of comorbidity

**Figure 2:**
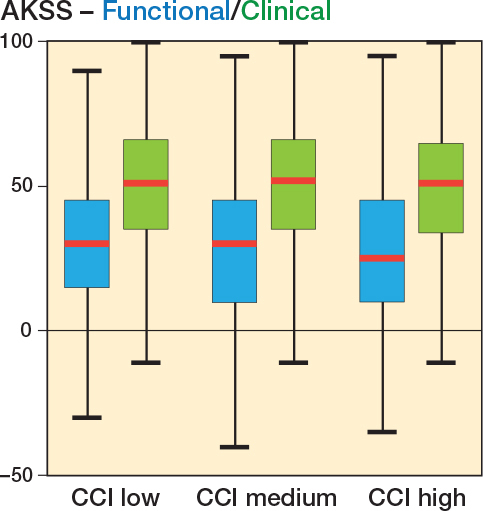
Box plot of median change in AKSS in functional (blue) and clinical (green) scores, respectively, for primary total knee arthroplasty. Red line is median, the box is IQR, and whiskers are 25th/75th percentiles ±1.5 x IQR, respectively. AKSS = American Knee Society Score. CCI = Charlson Comorbidity Index.

Patients undergoing TKA improved their functional and clinical AKSS from baseline to 1-year follow-up in all 3 CCI groups (Table 4 and Figure 2). In the functional AKSS, the mean change score decreased from 29 points (CI 29–29) in the CCI-low to 28 points (CI 27–29) in the CCI-medium to 26 points (CI 25–28) in the CCI-high group, respectively. In contrast, the mean change score in the clinical AKSS was 49 points (CI 48–49) in the CCI-low, 49 points (CI 48–50) in the CCI-medium, and 48 points (CI 46–50) in the CCI-high group, respectively. The mean change in both the functional and the clinical AKSS in all 3 CCI groups exceeded the MCID [15].

### Impact of comorbidity on changes in functional and clinical AKSS

**Figure 3:**
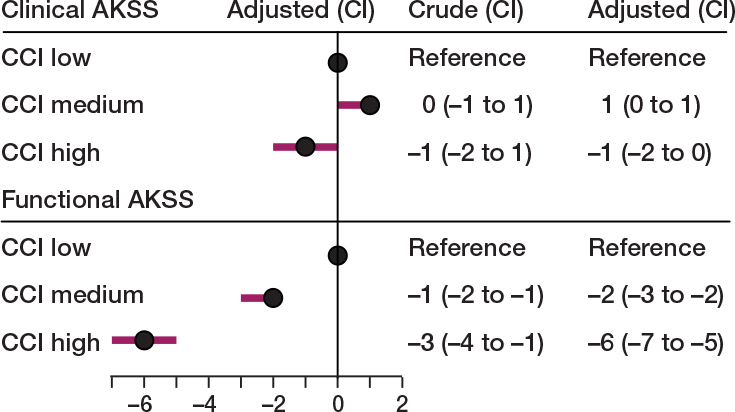
Forest plot of the mean difference in the adjusted^a^ association between multimorbidity and change in AKSS for patients undergoing primary TKA due to OA. ^a^ Adjusted for sex, age, weight, cohabiting, and baseline AKSS. For abbreviations, see Figure 2.

The improvement in both functional and clinical AKSS was significantly associated with the patient’s preoperative comorbidity status (Figure 3). The mean change score in the functional AKSS was –2 points (CI –3 to –2) in the CCI-medium and –6 points (CI –7 to –5) in the CCI-high groups compared with the CCI-low group. The mean change score in clinical AKSS in the CCI-medium and the CCI-high groups was 1 point (CI 0–1) and –1 point (CI –2 to 0), respectively, compared with the CCI-low group when adjusting for covariates sex, age, weight, cohabiting status, and baseline AKSS.

## Discussion

We aimed to investigate the impact of comorbidity on changes in functional and clinical knee scores 1 year after TKA using the AKSS.

We showed that, 1 year after TKA, functional and clinical AKSS across all CCI groups improved considerably and that the improvements were significantly associated with the patient’s preoperative comorbidity status. However, there were no clinically relevant differences in the improvement between the CCI groups as we had expected. The similar improvements between the CCI groups could be due to response-shift where patients in CCI-low undergo changes in their health-related quality of life after their TKA from baseline to 1-year follow up. Furthermore, threshold values for treatment success after TKA were achieved in the functional AKSS scores only for patients in the CCI-low and the CCI-medium groups, while patients in the CCI-high group did not meet these threshold values. The results suggest an association between comorbidity and functional AKSS after TKA.

Similar to our results, Elmallah et al. found a statistically significant change from baseline to 2- and 5-year follow-up in the functional and clinical AKSS in all CCI groups [3]. Comparable to our study, the differences in improvements between the CCI groups were not clinically relevant.

Several other studies have investigated the association between changes in pain or functional scores and comorbidity using measurement tools other than the AKSS. They have found similar results both for pain using the Western Ontario and McMaster Universities Osteoarthritis Index (WOMAC) and for function using, among others, the Knee Injury and Osteoarthritis Outcome Score (KOOS) and 6-Minute Walk Test (MWT) [6]. In line with the study by Elmallah et al., they found that patients with increased comorbidity had worse absolute scores for pain and physical function. However, they did not find an association between increased comorbidity and reporting of less improvement in pain or function after TKA. This suggests that an increased number of diseases may not limit improvement. However, by virtue of being associated with a worse “starting point,” they may be associated with a worse “ending point.”

Another study showed an increased severity of comorbidity indicates worse pain and functional outcome after TKA measured by the KOOS [5).

### Strengths and limitations

***Strengths.*** First, AKSS is a valid, reliable, and widely used functional outcome score for TKA [13,14]. Second, the CCI is commonly used in other studies of patients undergoing TKA [18]. A previous study based on data from the DKR used CCI as a proxy for the severity of comorbidity [19]. Third, applying multiple linear regression enables adjusting for known confounders such as sex, age, weight, cohabiting status, and baseline AKSS.

***Limitations.*** First, there was a ceiling effect of 27% in the functional AKSS and 10% in the clinical AKSS, which is common in AKSS [14,20], which may have led to an underestimation of the 1-year follow-up AKSS and be the reason for the similar improvements among the 3 CCI groups. Several studies have shown that ceiling effects are common in AKSS and may affect the validity of the results as the score is not sensitive enough to assess pain and function [14,20]. This results in failure to differentiate between patients with a more modest function and those with either expected or achieved higher levels after TKA [20]. The Tobit regression model might have eliminated some of the ceiling effects. Furthermore, AKSS is completed by the surgeon, which may lead to overestimating the changes, most likely being the same across the 3 CCI groups. Second, it is not mandatory to register the AKSS in the DKR, which resulted in incomplete information on AKSS in 78% of the patients who underwent TKA. However, the sensitivity analysis showed that patients without complete data on AKSS were comparable to those with a complete dataset, indicating that the data was missing by coincidence at random. Third, we were not able to adjust for confounders, such as pre-physical activity level; instead, adjustments were made on baseline AKSS as a proxy for pre-physical activity level. Furthermore, BMI is a known confounder for the association between comorbidity and functional and clinical AKSS after TKA [4-6]. Due to lack of data on BMI for the entire study population, we adjusted for weight as a proxy. Furthermore, the categorization of covariates may result in residual confounding in the categorized variables. Fourth, obese patients are not offered surgery to the same extent as people of normal weight, as weight loss is the first-line treatment for OA. It is reported in the literature that obese people have more severe comorbidity than normal-weight people [21], which might lead to selection bias. Thus, obese patients with very high comorbidity are not included. Finally, the concept of comorbidity is difficult to evaluate adequately with the available measurement tools. Furthermore, the CCI contains information on 19 chronic diseases, including dementia, but not other mental conditions. Mental conditions have been associated with functional and knee impairment changes after TKA [22]. It is suggested in future studies to include both CCI and mental conditions when determining the burden of comorbidity.

### Conclusion

Improvements in functional and clinical AKSS in patients with OA undergoing TKA were significantly associated with preoperative comorbidity level, but differences were small and unlikely to be clinically relevant between CCI groups. Patients with high comorbidity have poorer functional and clinical AKSS before surgery, and even though they have similar improvement to patients with no comorbidities, they do not reach the threshold value for treatment success to the same degree.

### Perspective

Clinical guidelines do not account for comorbidity and a “one-size-fits-all” approach is thus currently applied. It may be important for the surgeon to inform patients of the possible influence of their comorbidity status on outcomes, to align patients’ expectations as only patients in the CCI-low and the CCI-medium groups reached the threshold value for treatment success for functional AKSS.

## Notes

KGI analyzed the data and drafted the manuscript. All authors participated in the design of the study, interpretation of the results, and approved the final manuscript.

Handling co-editors: Marianne Westberg and Robin Christensen

*Acta* thanks Annette W-Dahl and Tina Strømdal Wik for help with peer review of this manuscript.
